# Preparation and Characterization of Soybean Protein Adhesives Modified with an Environmental-Friendly Tannin-Based Resin

**DOI:** 10.3390/polym15102289

**Published:** 2023-05-12

**Authors:** Hanyin Li, Yujie Wang, Wenwen Xie, Yang Tang, Fan Yang, Chenrui Gong, Chao Wang, Xiaona Li, Cheng Li

**Affiliations:** 1 College of Forestry, Henan Agricultural University, Zhengzhou 450002, China; 2 College of Landscape Architecture and Art, Henan Agricultural University, Zhengzhou 450002, China; 3 College of Material Science and Engineering, Nanjing Forestry University, Longpan Road 159, Xuanwu District, Nanjing 210037, China

**Keywords:** tannin, adhesive, soybean protein, plywood, wet shear strength

## Abstract

Soybean protein-based adhesives are limited in their application due to their poor wet bonding strength and poor water resistance. Herein, we prepared a novel, environmentally friendly soybean protein-based adhesive by adding tannin-based resin (TR) to improve the performance of water resistance and wet bonding strength. The active sites of TR reacted with the soybean protein and its functional groups and formed strong cross-linked network structures, which improved the cross-link density of the adhesives and then improved the water resistance. The residual rate increased to 81.06% when 20 wt%TR was added, and the water resistance bonding strength reached 1.07 MPa, which fully met the Chinese national requirements for plywood (Class II, ≥0.7 MPa). SEM observations were performed on the fracture surfaces of all modified SPI adhesives after curing. The modified adhesive has a denser and smooth cross-section. Based on the TG and DTG plots, the thermal stability performance of the TR-modified SPI adhesive was improved when TR was added. The total weight loss of the adhesive decreased from 65.13% to 58.87%. This study provides a method for preparing low-cost and high-performance, environmentally friendly adhesives.

## 1. Introduction

The production of traditional aldehyde adhesives, based on petroleum as the raw material, is hampered by their non-renewability and environmental and human health hazards [1], so the search for effective and environmentally friendly biobased adhesives [2] has become an urgent research goal.

Agroforestry biomass resources, such as lignin [3], tannins [4], and plant proteins [5] have been used as raw materials in the production of adhesives for coatings, packaging, and furniture [6]. Lignin is a biopolymer with a three-dimensional network structure, the second largest biomass resource in the plant kingdom after cellulose, which has an aromatic ring structure, with aliphatic and aromatic hydroxyl, and quinone groups. Lignin is widely used as a material in wood adhesives. It can be modified by enzymatic hydrolysis and acid catalysis [7]. Relevant studies have shown that adding lignin to polyurethane adhesives can also effectively improve adhesives’ peel resistance, adhesion, and filling properties [8]. Tannins mostly exist in plant bark or fruit as a polyphenolic compound. In recent years, various plant tannins, such as myrtle bark in the Brazilian Amazon [9] and Eucalyptus blue bark in Algeria [10], have been extracted to produce adhesives and these adhesives have good performance. In addition, using other biomass resources in adhesives production has shown good results, such as preparing biobased adhesives by co-blending tannins and lignin [11]. Among these resources, soybean protein has great potential as a raw material for synthesizing wood adhesives due to its renewable, non-toxic, and degradable properties [12,13]. The structure of soybean protein is a highly ordered spherical structure formed by the interaction between polypeptide chains through van der Waals forces, hydrogen bonds, and other stable multi-level structures. However, hydrophilic groups outside and hydrophobic groups wrapped inside result in poor adhesive performance and poor water resistance [14]. Therefore, modifying the soybean protein structure so that hydrophobic groups and other active functional groups can be exposed is a good strategy to improve the bonding strength and water resistance of soybean protein-based adhesives [15,16,17,18].

Currently, the modification methods commonly used to enhance the properties of soybean protein-based adhesives are acid-base, urea, surfactant, enzymatic, cross-linking, and grafting [6,19,20,21,22,23]. Alkali modification [19] increases the water resistance by changing the pH value and thus denaturing the protein, which exposes the non-polar groups. However, the protein hydrolysis increases under strong alkali conditions, resulting in lower molecular weight, weaker intermolecular interactions, and lower solid content. Furthermore, alkali modification has a detrimental effect on the adhesive’s mechanical properties and requires the addition of cross-linkers to enhance it. Carboxylic acids and calcium ions can improve protein-based adhesives’ physicochemical, thermal, and mechanical properties [20]. Cross-linking modification involves the active functional groups on the side chains of amino acids, such as amino, carboxyl, and hydroxyl, which can react with the cross-linking agent to form a tightly cross-linked network structure. This reduces the ability of water molecules on the adhesive to invade the adhesive system and improves the performance of adhesives. Epoxy compounds, anhydride compounds, and resin polymers are the most commonly used cross-linkers [24]. Modifying soy protein adhesives with melamine/epichlorohydrin prepolymer (MEP) can improve the water resistance of adhesives [21]. Multiple epoxy groups in MEP interact with reactive groups on protein molecules to form a tighter cross-linked network, and enhance the heat resistance of the adhesive as a rigid structure. Moreover, the rigidity of the adhesive increases, which further improves the water resistance. Modifying soy protein adhesives using grafting of hydrophilic groups on bisphenol A (E44) followed by phase change treatment with oil-in-water emulsions as cross-linkers can provide multiple stable cross-linking networks for soy protein molecules and improve the thermal stability and water resistance of the adhesives [22]. A soy glycoside molecule with two phenolic hydroxyl groups, grafted with epichlorohydrin, adds to a soy protein adhesive’s water and mold resistance [23]. While these cross-linking agents are effective in enhancing the performance of soybean protein-based adhesives, the amount of cross-linkers used in the modification process is often substantial. The synthetic raw materials for these cross-linkers are derived from fossil fuel-based resources, resulting in high synthesis costs and, ultimately, higher production costs [25]. Using biomass materials as cross-linking agents to modify protein-based adhesives is more in line with today’s environmentally friendly adhesive concept. It can greatly broaden the multi-functional utilization of biomass materials.

Tannins are low-cost natural polyphenolic biomass materials [26] containing large amounts of catechol/phthalate units that can react with the active function of soybean proteins through various non-covalent bonding [27,28]. The ortho- and para-positions of the benzene ring in the tannin structure have strong reactivity and can usually react with other active substances to form polymers. This has attracted research and the application of tannins in the wood adhesive industry has gradually increased. Kaiwen Chen [29] prepared hydrogel binders by adding tannic acid (TA) to a covalent network of polyethylene glycol diacrylate (PEGDA). Its presence promoted wet adhesion to various substrates by forming a common strong non-covalent bond, providing hydrophobicity and the possibility of reversible cross-linkers within the binary network to improve the mechanical properties of the gel. Xian-hui Shao [30] used three naturally occurring organic acids, namely tannins, lipoic acid, and phytic acid, to construct a novel adhesive gel for epidermal tissue dressing. The hydrogel can be synthesized under mild conditions, has good stability under continuous water immersion, and has good antibacterial activity. Abbas Hasan Faris [31] developed a green adhesive based on renewable lignin, tannin, and polyethyleneimine (PEI) to improve the water resistance of lignin/tannin adhesives; the improvement was probably due to the further reaction of the amino group in PEI with the catechol portion of lignin and tannin. This increased the cross-linked bonds in the resin, resulting in improved tensile strength, water resistance, and thermal stability. Abdelghani Boussetta [32] extracted proteins from shrimp waste, configured an adhesive with corn-starch-mimosa tannin (CSMT) at different CMST/P (*w*/*w*) ratios, and showed that corn starch, mimosa tannin, and proteins react through hydrogen bonds formed by hydrophilic groups, resulting in a more hydrophobic surface. Xianmou Fan [33] used TA as a chemical cross-linking agent and a provider of catechol structure to prepare a hydrogel, which has high strength and toughness through the network structure formed with kaolin.

This work synthesized tannin-based resin (TR) using valonea tannin as a raw material and then added it to a soybean protein adhesive to produce a plywood adhesive. The tannin-based resin was prepared by replacing part of the phenol structure with the biomass material tannin, which improved the performance of the adhesive by cross-linking with soy protein. This process would reduce the consumption of fossil fuel-based raw materials in plywood production. The properties of the adhesives were characterized, such as adhesive viscosity, functional groups of the cured adhesives, thermal stability, and thermal properties. In addition, the bonding strength of plywood specimens fabricated with the produced adhesives was evaluated.

## 2. Materials and Methods

### 2.1. Materials

Soybean protein isolate (SPI) was obtained from Shandong Yuwang Grain and Oil Company, (Jinan, China) (200 mesh). The components of SPI were soybean protein isolate (96.58%), sugars (1.24%), moisture (2.86%), and fat (0.16%). Hydrolyzed tannin (purity 68%), phenol (99%), and formaldehyde solution (37%) were purchased from Wuhan Huaxiang Kejie Biotechnology Co. (Wuhan, China), Tianjin Damao Chemical Reagent Factory (Tianjin, China), and Yantai Shuang Shuang Chemical Co. Ltd. (Yantai, China), respectively. Poplar (*Populus* sp.) veneer was provided by Shandong Kaiyuan Wood Industry Co., Ltd. (Linyi, China). with a moisture content of 8%. The size of the veneer was 400 × 400 × 3 mm.

### 2.2. Synthesis of Tannin-Based Resin

In the first step, 20 g of tannin, 80 g of molten phenol, 64 g of sodium hydroxide solution (40 wt%), and 70 g of distilled water were added into a flask equipped with a condensing tube and a magnetic stirring device, and then heated to 60 °C in the water bath. Stirring of the mixture and increasing the temperature of the water bath continued until it reached 85 °C. A 50% formaldehyde solution was added to the flask and then reacted for 30 min at 85 °C. In the second step, 54 g of formaldehyde solution (37 wt%) was added and stirred for 50 min at the same temperature. The third step involved repeating the second step. Finally, the reaction products were rapidly cooled to 40 °C, and the tannin-based resin was obtained. The resulting TR had a solid content of 36.2% at 20 °C and a pH value of 10.76.

### 2.3. Soybean Protein Isolate-Based Adhesives

As shown in Table 1, 20 g soybean protein isolate was mixed with 80 g water at room temperature and rapid stirring continued for 9 min until it was completely dispersed and homogeneous to obtain the SPI adhesive. Then, based on the weight of SPI adhesive, 5, 10, 15, 20, and 25 wt% of TR was added and stirred well to obtain TR-modified SPI adhesives. The modified adhesives were denoted as 5 wt%TR, 10 wt%TR, 15 wt%TR, 20 wt%TR, and 25 wt%TR.

### 2.4. Performance of Adhesives and Plywood

A sheet of three-layer plywood was prepared from poplar veneer using a single-sided manual gluing process, where the middle and bottom layers were glued on one side with an application rate of about 145~150 g/m^2^. After gluing, the plywood was assembled so that the wood grain of each layer of veneer was perpendicular to the next and then pressed using a Universal testing hot press (R50t, Suzhou Xinxieli Co., Ltd., Suzhou, China). The process was as follows: the plywood sample was pressed at 1.1 MPa for 6 min at 125 °C and then placed in a normal environment for 24 h, and its properties were measured.

Determination of the solid content of the adhesive samples was assessed via the dry weighing method based on the weight difference before and after drying in the oven at 120 °C. First, the glass tray was dried in the oven at 120 °C until it reached a constant weight (*M*_1_) and recorded. Then, 4 ± 0.1 g of adhesive was poured into the glass tray, and the weight *M*_2_ was recorded. The adhesive was dried in the oven at 120 °C until it reached a constant weight, and the weight *M*_3_ was recorded.

$$Solid\;content(\%)=\frac{M_{3}-M_{1}}{M_{2}-M_{1}}\times 100\%$$

**Figure 1:**
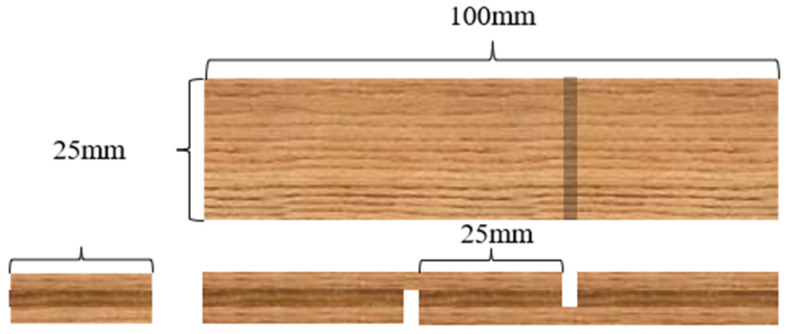
Plywood test specimen.

The bonding strength of the plywood was tested according to the relevant national standard GB/T 17657-2013. As shown in Figure 1, the pressed plywood was cut and processed into test pieces (10 cm × 2.5 cm), which were then immersed in boiling water for 3 h, removed, and cooled for 10 min. The gluing strength was determined using an electronic universal testing machine (WDW-20). The gluing strength was calculated using the following formula:

$$Bonding\;strength(\mathrm{MPa})=\frac{P_{max}}{b\times l}$$

where *P_max_* is the maximum breaking load (N); *b* is the section width (mm); and *l* is the section length (mm). The final strength values were taken as the average of 8 specimens.

The water resistance of the adhesive samples was assessed with the method used by Li et al. [24]. The samples were dried to a constant weight in an oven set at 120 °C, ground into powder, passed through a 200-mesh sieve, and weighed at a certain mass (*M*_1_) in a filter paper bag of mass *M*_2_. The filter paper bag was then immersed in distilled water at a temperature of 20 °C, with the water being replaced every 20 min. After 1 h of soaking, the filter paper bag was dried to constant weight in an oven and weighed as *M*_3_. The test was conducted three times and averaged. The residual rate is calculated concerning the following equation:

$$Residual\;rate=\frac{M_{3}-M_{2}}{M_{1}}\times 100\%$$

### 2.5. Fourier Transform Infrared (FTIR) Spectroscopy

All adhesives were freeze-dried with a freeze-dryer (FD-2A) until the moisture was completely removed. The dried adhesive powder (200 mesh) was uniformly blended with potassium bromide (KBr) in a mass ratio of 1:80 and then pressed into tablets to be analyzed using IR spectroscopy. The experimental parameters of the Fourier transform infrared spectroscopy (Nicolet iS10, Madison, WI, USA) were set as follows: wavenumber was from 500 to 4000 cm^−1^ and scanning 32 times under the resolution of 4 cm^−1^.

### 2.6. X-ray Diffractometer (XRD) Testing

Cured and dried adhesive samples were ground into powder (particle size around 45 µm) and then tested on an X-ray diffractometer (D8 Advance, Panalytical, Holland). Testing conditions were set as follows: the test target was copper; the scanning speed and angle ranges were 2°/min and 5–60°, respectively. The crystallinity of the adhesive samples was calculated following the report by Pang et al. [22].

### 2.7. Measurement of Thermal Properties

All adhesives were completely cured and dried in an oven at 125 °C for 3 h, and then ground in an agate mortar to 200 mesh. The samples (8~10 mg) were tested using a thermogravimetric analyzer (Q5000IR, TA Instruments, USA). The test conditions were: temperature from 30 °C to 600 °C, with a heating rate of 10 °C/min, N_2_ environment.

### 2.8. Scanning Electron Microscopy (SEM)

The cured SPI adhesives were crushed to obtain a smooth and flat fracture surface. The fracture surface of the resin was sprayed with a gold coating and then observed using an SEM (SU8010, Hitachi, Japan), and at least three images were taken for each sample.

## 3. Results and Discussion

### 3.1. Effect of Solid Content

**Figure 2:**
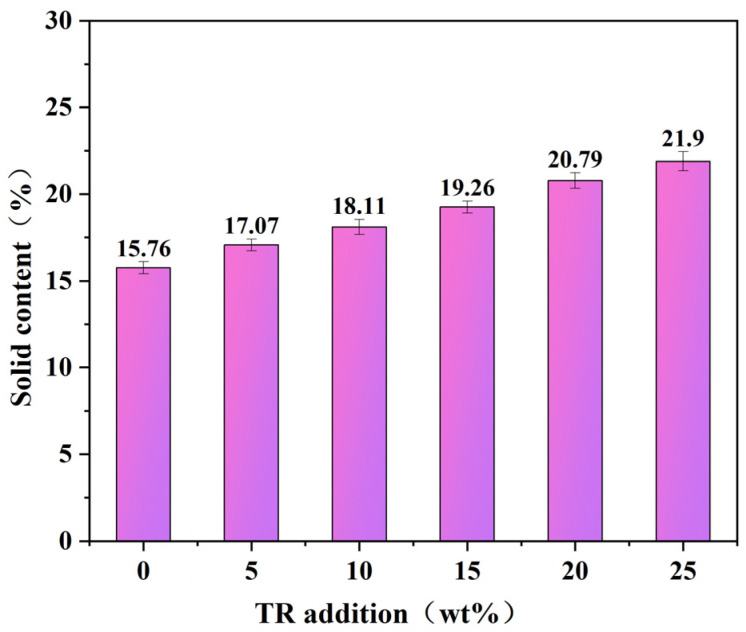
The solid content of SPI adhesives with different weight ratios of TR.

The solid content of wood adhesives has an important impact on the performance of plywood, influencing the formation of the glued layer on wood panels [34]. The solid content of modified SPI adhesives with different TR concentrations is shown in Figure 2.

Figure 2 shows that the solid content of the pure SPI adhesive is 15.76%. This low solid content was related to the ratio of ingredients used in the adhesive preparation. The high protein content of soybean protein powder increased intermolecular friction, resulting in high viscosity and poor fluidity of the adhesive, with a paste-like appearance. Using the existing ingredient ratio to increase the quantity of soybean protein powder will change the adhesive from a paste to a dry powder, preventing it from adhering to the wood panel. Adding additives with high solid contents improved the solid content of soybean protein-based adhesives. As shown in Figure 2, the solid content of TR was 36.2%, and when 5 wt% TR was added, the solid content of soybean protein adhesive increased to 18.11%. This is consistent with the expected results as the solid content of the soybean protein adhesive increased gradually with the increased quantity of TR; at 25 wt% TR, the result increased from 15.76% to 21.90%. The increase in the solid content of modified SPI adhesive would reduce the water evaporation and elimination time during the pressing of plywood, the water aggregation in the core of the board will reduce, and the production efficiency will increase.

### 3.2. Bonding Strength Analysis

**Figure 3:**
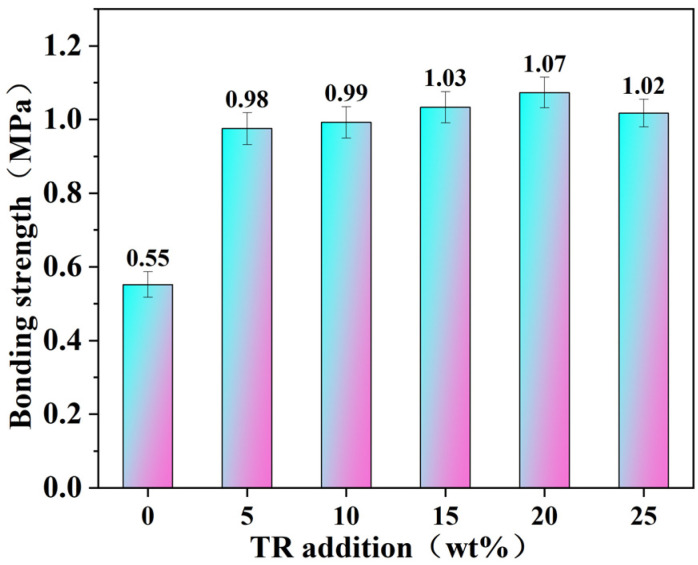
Wet shear strength SPI adhesives with different ratios of TR.

The wet bonding strength of cross-linked modified soybean protein isolate-based (SPI) adhesives with different TR additions is shown in Figure 3. Pure SPI adhesive has a bonding strength of 0.55 MPa, which is lower than the Chinese national standard plywood requirement (class II, ≥0.7 MPa). When soybean protein is dissolved in water, its molecular chains may fold over each other. During the hot pressing process, the molecular chains congregate and twist with one another, generating a force that bonds the boards together [35,36,37,38]. The introduction of the TR cross-linking agent significantly enhanced the bonding strength of the modified SPI adhesives, resulting in pressed plywood that meets the Class II plywood requirements. The bonding strength of 5 wt% TR was 0.98 MPa, 78.2% higher than pure SPI adhesive. As the quantity of TR increased, the bonding strength of the modified SPI adhesive gradually increased until 1.07 MPa was achieved with 20 wt% TR. Wu et al. [39] employed phenol-formaldehyde and phenol-glutaraldehyde resin to enhance and modify their soybean protein adhesive. Under the conditions of hot pressing temperature of 180 °C, processing time of 5 min, and pressure of 1.5 MPa, the bonding strength of plywood was 0.86 and 0.88 MPa, respectively. Lei et al. [40] used epoxy resin (EPR), melamine-formaldehyde resin (MF), and EPR-MF resin to promote and modify soybean protein. The bonding strength was 0.46, 0.94, and 1.08 MPa, respectively. The plywood was pressed under pressure of 2 MPa at 160 °C for 8 min. Compared with the above investigations, the TR-modified SPI adhesives in this study has advantages such as good bonding strength, better water resistance, low cost, and mild operations (temperature, pressure, and time) for the hot pressing process.

TR enhanced the bonding strength and water resistance of SPI adhesive mainly due to two reasons. (1) The sodium hydroxide in TR can denature the structure of SPI and destroy the α-helical and β-folded structures in its secondary structure, causing the molecular protein chains to unfold and the internal hydrophobic groups to appear, which improves the water resistance of the adhesive. (2) FTIR analysis reveals that TR may interact with proteins, causing some active groups on the side chains of amino acids to react and form a cross-linked structure with high strength and density, further improving the adhesive’s bonding performance. This result agrees with the findings of the residue rate test analysis.

### 3.3. Residue Analysis

**Figure 4:**
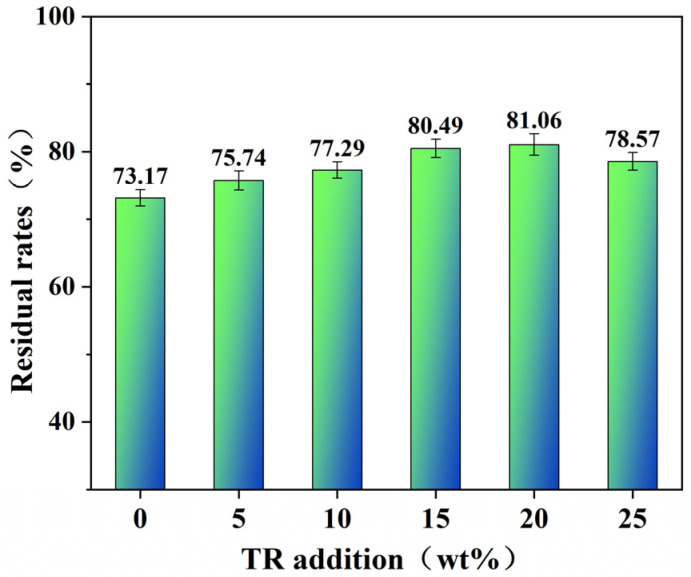
Residual rate of SPI adhesives with different ratios of TR.

The water resistance of the adhesive has an important impact on the water resistance of the plywood. The water resistance of the adhesive is related to the cross-linking density inside the adhesive. When an adhesive with a low degree of cross-linking is soaked in water, it is easily eroded by moisture because as the intermolecular force decomposes, the adhesive decomposes and dissolves in water. In contrast, adhesives with a high degree of cross-linking have a network cross-linking structure with high bond energy strength between molecules, which can effectively reduce the invasion of water, reduce solubility, and improve water resistance. The adhesive soaking water residue test can characterize the degree of cross-linking of the adhesive, and the test results are shown in Figure 4.

The residual rate of pure soybean protein isolate-based adhesive was 73.17%; soybean protein isolate contains many polar groups with good water absorption, and the curing process of soybean protein isolate has no chemical reaction. The intermolecular force is mainly hydrogen bonds that, when encountering water, are easily broken. Protein molecules are hydrolyzed to form small aggregates, resulting in mass loss, so the residue rate was the lowest. After the addition of TR resin, the residue rate of the adhesive improved, indicating that the soybean protein isolate-based adhesive modified by TR resin cross-linking had a good degree of cross-linking. TR resin contains sodium hydroxide, which is alkaline and can cause damage to the molecular structure of the protein, denaturing the protein, opening and stretching the molecular chain of the protein, and exposing the hydrophobic group inside to improve the water resistance of the adhesive. Moreover, because the active group on the molecular protein chain reacts with TR resin to form a cross-linked and interlocking structure, it can resist the invasion of water well and improve the residue rate. With increasing TR resin additions, the residue rate of the modified adhesive continued to increase. When the TR resin addition amount was 20 wt%, the highest residue rate of the adhesive was 81.06%. When the TR resin addition amount was further increased to 25 wt%, the residue rate of the adhesive decreased to 78.57%, indicating that the cross-linking density of the adhesive decreased. This is because there is an excess of TR resin, and part of it does not cross-link with soy protein but instead undergoes its self-condensation reaction, which reduces the crosslinking density of the adhesive.

### 3.4. Characterization of the Structure of TR and Different Modified SPI Adhesives

**Figure 5:**
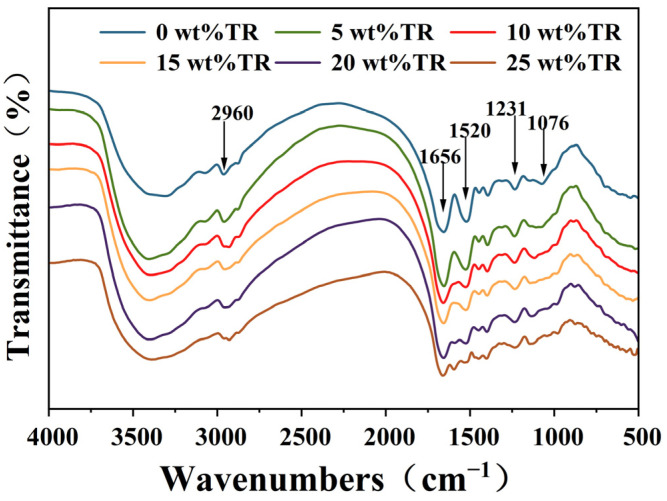
FTIR spectra of different SPI adhesives modified with different TR additions.

The infrared spectra of the cross-link modified SPI adhesives with different TR additions are shown in Figure 5. The main active functional groups on the side chains of amino acids, which are constituents of soybean protein, are amino (-NH_2_), carboxyl (-COOH), and hydroxyl (-OH). For the pure soybean protein isolate-based adhesive, the absorption peaks at 3288~3421 cm^−1^ are caused by -NH and -OH stretching vibrations [41], and the peak at 2959 cm^−1^ belongs to methylene -CH_2_ in TR [42]. The typical amide I (C=O), II (N-H), and III (C-N and N-H) bonds in the SPI structure correspond to the peak of 1656, 1520, and 1234 cm^−1^, respectively [43,44]. The peaks at 1076 cm^−1^ are attributed to C-O stretching vibrations, and COO- stretching vibrations were observed at 1392 cm^−1^ [45].

As seen in Figure 5, the main characteristic peaks of the soybean protein isolate-based adhesive did not disappear when TR was added, indicating that the primary structure of the protein was preserved. However, the absorption peaks of the amide II and III bonds of the soybean protein isolate-based adhesive decreased. The absorption peak at 1392 cm^−1^ also appeared to decrease due to the reaction between TR and the functional groups on the soybean protein isolate. This indicates that the TR reacted with amino, carboxylic acid, and hydroxyl groups on protein molecules to form a cross-linked structure. Moreover, with increasing TR additions, the intensity of these peaks gradually becomes weaker. When the addition of TR was 25 wt%, the soybean protein isolate-based adhesive showed a new peak at 1595 cm^−1^. This peak corresponds to the stretching vibration of the conjugated double bond of the benzene ring (-C=C-), caused by the excessive addition of TR, of which, some cross-linked with the soybean isolated protein, and the remaining part underwent a self-condensation reaction.

### 3.5. X-ray Diffraction (XRD) Analysis

**Figure 6:**
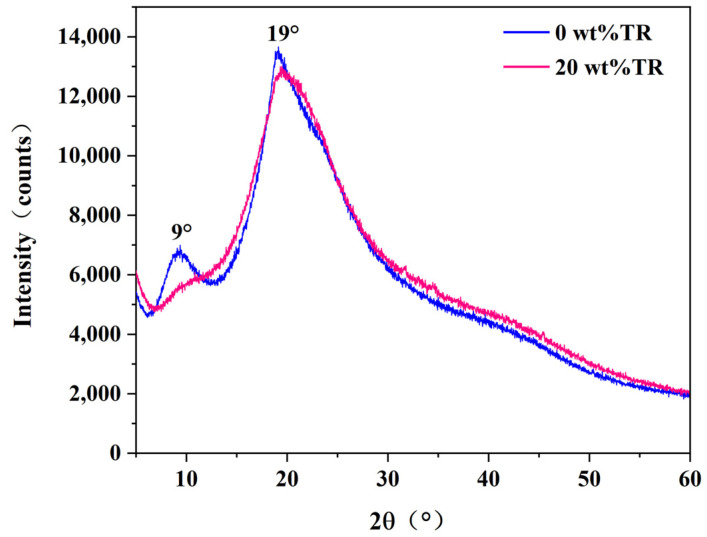
XRD spectra of adhesives without and with a 20 wt% TR.

Figure 6 shows the XRD patterns of the SPI adhesive and SPI-20 with a 20 wt% cross-linker. For the pure soybean protein isolate-based adhesive, there are two diffraction peaks near 2θ of 9° and 19°, which are characteristic peaks of the α-helical and β-folded structures in the secondary structure of the protein, respectively [46]. The soybean protein isolate-based adhesive without TR generates no chemical reactions during curing. It is a process of aggregation and entanglement between protein molecules; therefore, its molecular structure remains intact. When 20 wt% of TR was added, the diffraction peak near 2θ = 9° of the modified adhesive showed a broad and diffuse weak peak compared to the pure soybean protein adhesive, indicating that TR caused damage to the molecular structure of the protein [47].

The peak of the β-fold structure of soybean protein isolate-based adhesive was 2θ = 19.1°, and after adding 20 wt% of TR, the peak widened and shifted to 19.5°. This is because the TR disrupts the molecular structure of the protein and reacts with it by cross-linking [48]. The crystallinity of the soybean protein isolate-based adhesive without TR was 17.46%, and that of the modified adhesive decreased significantly to 14.47%. According to the analysis above, the curing of pure soybean protein isolate-based adhesive is a process of intermolecular entanglement and aggregation; hence, the protein molecular structure is completely preserved, and its crystalline area combines with intermolecular agglomeration. The reasons for the decrease in crystallinity of the SPI adhesives with the addition of 20 wt% TR can be attributed to the fact that TR is alkaline and can disrupt the secondary structure of the protein, causing it to be out of balance. The molecular chains are rearranged and dispersed.

### 3.6. Thermal Properties Analysis of Different SPI Adhesives

**Figure 7:**
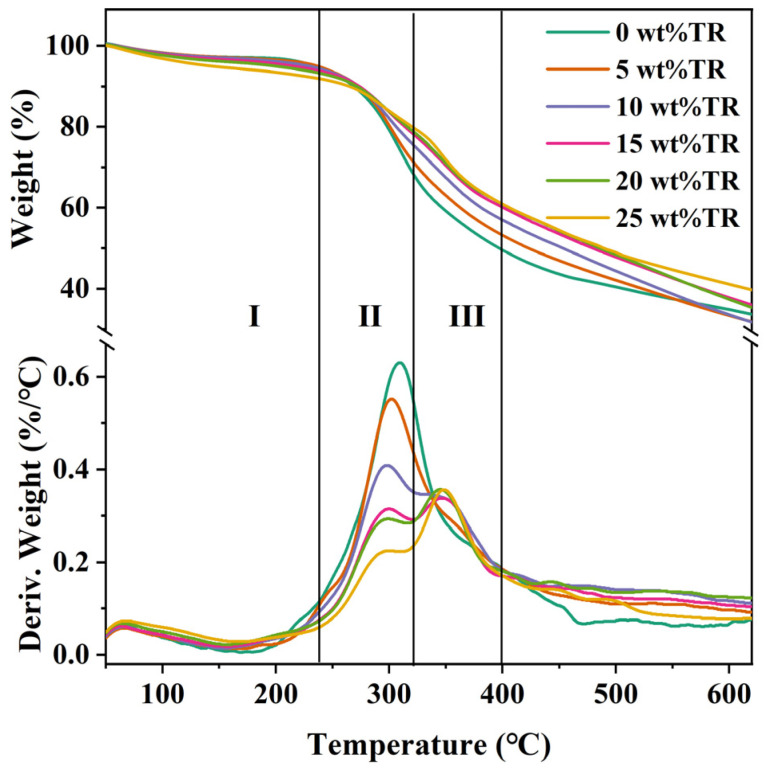
TG and DTG curves of SPI adhesives modified with different TR additions.

The thermal properties of the cross-linked modified SPI adhesives with different TR additions were investigated using thermogravimetric analysis (TG), and the results are shown in Figure 7. There are three stages of the thermal degradation process of all adhesives. The first stage occurred at 60~240 °C, when the mass loss was mainly caused by the decomposition of the four-level structure of SPI and water evaporation [49,50]. The second stage occurred at 240–320 °C, when the weight reduced significantly, mainly due to the degradation of SPI. During this stage, some unstable chemical bonds in the adhesive were broken [51,52]. The third stage occurred at a temperature of 320 to 400 °C, which was caused by the degradation of the cross-linked structure of the adhesive. During this stage, the breakage of the covalent peptide, which bonds to the amino acid residues of the SPI, resulted in mass loss [53].

Table 2 lists the temperatures (T_max_) corresponding to the maximum degradation rates of the adhesives in different degradation stages and the total weight loss. From the TG and DTG curves of Figure 7, all adhesives showed a smooth reduction trend in weight loss and degradation rates in the first stage. For the second stage, the pure SPI adhesive had the maximum degradation peak at T_max_ = 307.87 °C because its curing process produces no chemical reactions and a low degree of cross-linking, and the intermolecular force is mainly hydrogen bonding, which is easily broken and decomposed when subjected to high temperatures. After TR was introduced into the SPI adhesive, the degradation peak of the modified adhesive decreased with increasing TR additions, indicating that its cross-linking density was improved. In the final stage, 25 wt% TR showed the maximum degradation rate at T_max_ = 346.67 °C. This is caused by the excess TR, of which some cross-linked with SPI, and the rest underwent a self-condensation reaction, resulting in a cross-linked interlocking structure in the system. At this stage, the systemic skeleton degradation of the adhesive was not only the decomposition of the peptide chain but also the mass loss due to the water molecules generated by the methylene breakage and hydroxymethyl condensation that occurred due to the self-condensation of TR.

When the addition of TR cross-linker was increased from 0 wt% to 25 wt%, in 5 equal increments, the total weight loss of the adhesive was 65.13%, 66.62%, 66.40%, 62.61%, 62.19%, and 58.87%, respectively. Based on the TG and DTG plots, the thermal stability performance of the TR-modified SPI adhesive was improved when TR was added.

### 3.7. Scanning Electron Microscope Analysis

**Figure 8:**
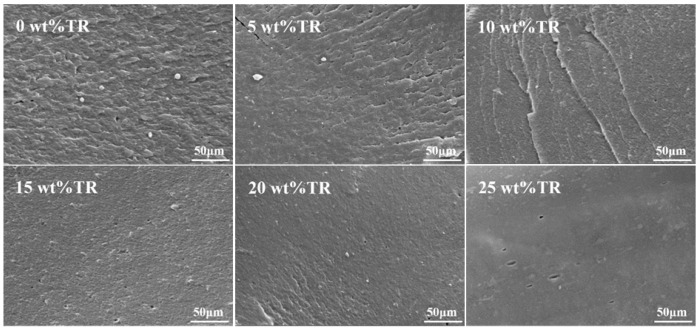
SEM images of adhesives with different TR additions.

SEM observations were performed on the fracture surfaces of all modified SPI adhesives after curing. From Figure 8, we can see that the fracture surface of the pure SPI adhesive was rough, loose, and had voids, since no intermolecular chemical reaction occurred during curing. Therefore, there was no formation of a densely cross-linked structure between the molecules. This surface structure easily swells and fractures when in contact with water through its voids, resulting in an adhesive with less water resistance. On the other hand, the modified adhesive has a denser and smooth cross-section, caused by the reaction between TR and the active groups (amino, carboxyl, hydroxyl, etc.) of SPI, which formed a tightly cross-linked structure that significantly limited the invasion of water and improved the water resistance of the adhesives.

## 4. Conclusions

In this study, a low-cost and environmentally friendly TR, used as a cross-linking agent, enhanced the soybean protein adhesive’s performance. TR effectively improved the SPI-based adhesive’s bonding performance and water resistance of plywood. The main reasons are three-fold. (1) FTIR results showed that the absorption at 1520 and 1234 cm^−1^ became weaker with the addition of TR, indicating that TR reacted with the reactive groups of soybean protein (-NH_2_, -COOH, -OH, etc.) and formed a tightly cross-linked structure. (2) The XRD results showed that the α-helical peak in the protein secondary structure was disappearing. The β-folded peak was displaced after adding TR, indicating that TR also reacted with SPI to increase the cross-link density in the adhesive system, reducing the crystallinity of the pure SPI adhesive from 17.46% to 14.47% after adding 20 wt% of TR. (3) The introduction of TR increased the SPI adhesive’s solid content and residual rate. The improved water resistance, solid content, and cross-linking density of the modified SPI adhesives resulted in significantly improved bonding strength of the fabricated plywood specimens (≥0.7 MPa), with the highest bonding strength of 1.07 MPa in the 20 wt% TR-modified SPI adhesive. The plywood prepared with TR-modified SPI adhesives exhibits excellent water resistance and bonding strength. Compared to chemically crosslinked such as isocyanates, epoxy resins, hyperbranched polymers, dopamine, etc., the TR-modified SPI adhesives have the advantages of environmental protection, low cost, and simple operation. At the same time, mild operations (temperature, pressure, and time) are required for hot pressing. Therefore, this adhesive has great application prospects in wood-processing industries, such as plywood and blockboard production.

## Figures and Tables

**Table 1:** TR cross-linking-modified SPI adhesive ingredient ratio.

Adhesive	SPI (g)	Water (g)	TR (g)
0 wt%TR	20	80	0
5 wt%TR	20	80	5
10 wt%TR	20	80	10
15 wt%TR	20	80	15
20 wt%TR	20	80	20
25 wt%TR	20	80	25

**Table 2:** Thermal properties of different adhesive samples.

TR Addition (wt%)	The First Stage (°C)	The Second Stage T_max_ (°C)	The Third Stage T_max_ (°C)	Total Weight Loss Rate (%)
0	—	307.87	—	65.13
5	—	300.6	—	66.62
10	—	295.73	346.22	66.40
15	—	298.58	343.40	62.61
20	—	297.6	343.96	62.19
25	—	296.19	346.67	58.87

## Data Availability

The data presented in this study are available on request from the corresponding author.
